# Venous gangrene and cancer: A cool look at a burning issue

**DOI:** 10.1186/1477-7800-4-7

**Published:** 2007-03-27

**Authors:** Khalid A Osman, Mohamed H Ahmed, Samir A Abdulla, Tim E Bucknall, Colin A Rogers

**Affiliations:** 1 Department of Surgery, Queen's Hospital, Burton Hospitals NHS Trust, Staffordshire, UK; 2 Department of Chemical Pathology, Southampton General Hospital, Southampton, UK

## Abstract

Venous gangrene (VG) is defined as a clinical triad of skin necrosis and discolouration, documented evidence of venous thromboembolism (VTE) and presence of palpable or doppler- identifiable arterial pulsation. Venous gangrene is rare condition which is associated with poor prognosis in cancer patients. The pathogenesis of VG is multifactorial and could paradoxically be due to warfarin treatment. Heparin Induced Thrombocytopenia (HIT) associated venous gangrene develops when heparin therapy is discontinued and warfarin therapy initiated or continued.

It has been reported that the presence of anticardiolipin antibodies appears to double the risk of thrombo-embolic events in cancer patients in comparison with those who are anticardiolipin antibody negative. The presence of anticardiolipin antibodies is therefore a warning sign for venous gangrene in cancer patients. Hypercoagulable state associated with malignancy, cancer treatment, prolonged immobilisation, surgical operations and metabolic syndrome are all associated with increased risk of VTE and VG.

The current evidence suggests that cancer patients are at increased risk from recurrent venous thrombosis and venous gangrene, and LMWH provides potential promise as a safe and effective measure in the management of such patients.

## Background

Venous gangrene (VG) is a rare condition in association with malignancy but carries a grave prognosis [1]. Venous gangrene does not occur in isolation of VTE. Patients with cancer have long been recognised to be at a high risk of venous thromboembolism, nevertheless the condition remains under-diagnosed and under-treated in these patients. In consequence, the morbidity and mortality due to thromboembolism remains unacceptably high. Furthermore, the management of such patients in the presence of malignancy is complex, due to the effects of cancer itself and its treatments [1,2].

Interestingly, VG could paradoxically be due to warfarin treatment in association with decreased level of protein C [3]. The epidemiology, pathogensis and management of cancer-related venous gangrene are discussed in this review.

## Incidence

Currently the incidence of VG in association with cancer is not well established. However, there are a few reported cases in the literature showing that VG is always in association with venous thrombo-embolism (VTE). The annual incidence of VTE in a cancer population is 500 in 100,000 (one in two-hundred) in comparison with 117 in 100,000 in the general population [1,4]. Rates of VTE as high as 43% in patients with metastatic renal cell carcinoma receiving chemotherapy has been reported [5]. In an analysis of the autopsy records of 157 cases with carcinoma of the pancreas, venous thromboembolism was found in 50% of patients at post-mortem examination [6].

In their study of 1041 patients with solid tumours admitted to 3 major medical centres in the USA, Sallah et al found the highest rates of VTE in cases of advanced malignancies, renal carcinoma, pancreatic, gastric and brain tumours. Leading the view that mucin-producing tumours are most often strongly associated with the occurrence of venous thrombosis [7]. However, the most common malignancies associated with thrombosis are those of the breast, colon and lung, reflecting the prevalence of these malignancies in the general population [1]. Further research is needed to establish the incidence of VG in association with cancer.

## Pathogenesis

The pathogenesis of (VG) is obscure; however, venous gangrene does not occur in isolation of venous thromboembolism. Venous gangrene could paradoxically be due to warfarin treatment and develop when the international randomised ratio (INR) is above 6.0, therapeutic range (2.0–3.0). At this supra- therapeutic level of INR the level of protein C is markedly decreased but the thrombin-antithrombin complexes remain unexplainably high [3]. This profound disturbance in procoagulant-anticoagulant balance during warfarin treatment leads to progressive microvascular thrombosis secondary to acquired natural anticoagulant depletion during warfarin therapy. In addition, warfarin anticoagulation can cause paradoxical thrombotic events, particularly central skin necrosis of the breasts, abdomen and thighs in patients with congenital heterozygous protein C deficiency [8,9]. It has been postulated that warfarin-induced skin necrosis is caused by a transient prothrombotic state that results from a faster reduction in the level of the major natural anticoagulant factor (protein C; half-life, 6 hours) than in the level of the major procoagulant factor (prothrombin; half-life, 72 hours) [10].

Furthermore, in a study of 158 patients with heparin-induced thrombocytopenia (HIT), 8 patients developed acute venous limb gangrene after heparin therapy was discontinued and warfarin therapy either initiated or continued. In these 8 patients the INR level was at suprat-herpeutic [10,11]. HIT is caused by a platelet-activating, heparin-dependent IgG antibody and is an important cause of paradoxical arterial and venous thrombotic complications. It is suggested that a warfarin-induced failure of the protein C anticoagulant pathway to regulate the increased thrombin generation that occurs in patients with heparin-induced thrombocytopenia, that leads to venous thrombosis and gangrene[12-14].

Antiphosolipid antibodies may also be responsible for the increased venous thrombosis in cancer patients. Anti-phospholipids antibodies are mainly composed of the lupus anticoagulant and anticardiolipin antibodies. These antibodies predispose to thrombosis either by interacting with phospholipids in the platelets and the vascular endothelium or by inhibiting protein C activation and prostacyclin formation in the endothelial cells. It has been reported that the presence of anticardiolipin antibodies appears to double the risk of thrombo-embolic events in cancer patients in comparison with those who are anticardiolipin antibody negative (28% versus 14%) [1,15].

The hypercoagulable state associated with malignancy is thought to be due to: direct activation of clotting system by cancer cells, and indirectly by activation of platelets, monocyte and endothelial cells. Cytokines such as tumour necrosis factor-α (TNF-α) and interleukin-1β released by tumour cells activate the tissue factor (TF). This transmembrane protein which found only on fibroblasts of vascular adventitia and other stromal cells is also expressed on the surfaces of all solid tumour cells. It initiates coagulation by binding to activated factor VII. Cytokines increase the expression of TF and platelets activating factors (PAF) and decrease the expression of thrombomodulin and the endothelial cells protein C receptors. This imbalance in procoagulant-anticoagulant pathways leads to the hypercoagulable state associated with malignancy leading to the increased risk of thrombosis in cancer patients. In addition, decreased levels of antithrombin III, deficiency of protein C and S were reported with cancer [16-19]. Significantly, cancer treatment especially chemotherapy and hormonal therapy such as tamoxifen significantly contributes to the increased risk of thrombosis in cancer patients [20].

Immobilisation, prolonged bed rest, dehydration and vomiting also significantly increase risk of thromo-embolism [1,2]. In addition, patients undergoing surgery for cancer have a higher risk of postoperative deep vein thrombosis (DVT) than those having surgery for non-malignant disease. After surgery, cancer patients have twice the risk of DVT and over 3 times the risk of fatal pulmonary embolism (PE) compared with patients free of cancer [21,22].

Several studies have described the association between metabolic syndrome and the increased tendency towards hypercoagualtion in general population [23,24]. The clustering of insulin resistance, dysglycemia, dyslipidemia, hypertension and central obesity represent the major features of metabolic syndrome. The metabolic syndrome appears to affect between 10 and 25% of adult populations worldwide [25]. Recently, Kikura et al demonstrated that features of the metabolic syndrome are risk factors for perioperative arterial or venous thromboembolism events and subsequent death within 30 postoperative days in a total of 21,903 surgical patients followed for 11 years [26].

Taking all these factors into consideration, it is possible to postulate that the presence of metabolic syndrome, cancer and paradoxical effect of warfarin may enhance the process of DVT formation and this ultimately may have lead to venous gangrene in cancer patients (table 1).

**Table 1:** Predisposing factors for developing venous thrombosis and gangrene in cancer patients

• Supra-therapeutic level of INR ≥ 6.0
• *Acquired Protein C and protein S deficiency as result of warfarin treatment
• *Heparin induced thrombocytopenia, after initiating or continuing warfarin therapy
• *Cancers of the pancreas, lung, stomach and adenocarcinoma of unknown primary.
• *Positive anticardiolipin antibodies
• *Obesity and metabolic syndrome
• Cancer treatment (chemotherapy or hormonal e.g. tamoxifen)
• Surgical operations

*Risk of recurrent thromboembolism

## Prevention and management of venous gangrene

The prevention of venous gangrene is similar to that of VTE. Attention to good nutrition, hydration and early ambulation are of paramount importance in cancer patients (table 2). Use of prophylactic LMWH or unfractionated heparin (UFH) perioperatively together with graduated compression stocking reduces the incidence of postoperative DVT significantly. LMWHs are convenient, efficacious and safe compared with UFH and coumarin derivatives and are becoming the anticoagulant class of choice in surgical and medical oncology patients. Extending prophylaxis with LMWH beyond hospitalization after curative open surgery for abdominal or pelvic cancer for three weeks to four weeks in cancer patients has shown to reduce the rate of VTE by up to 62% in one study and to none in another study [27,28]. (Table 2)

**Table 2:** Prevention of VTE and VG in cancer patients

**General measures**: attention to good nutrition, hydration and mobilisation
**Physical/mechanical measures**: TEDS (Thromboembolic Deterrent Stockings) And intermittent pneumatic compression Stocking during surgical operations
**Pharmacologic measures**: LMWH, UFH Starting warfarin at low dose simultaneously with LMWH in case of established VTE

In contrast, Andtbacka et al, in their retrospective study of 3898 patients with breast cancer, who underwent 4416 surgical procedures for different stages of breast cancer concluded that the risk of VTE following breast cancer surgery is rare (rate of 0.16 per procedure). This lower rate of VTE was achieved by adherence to their clinical pathway using mechanical antithrombotic devices and early ambulation in the postoperative period, without the need for systemic VTE prophylaxis in the form of LMWHs [29].

The conventional treatment of VTE is to start with therapeutic dose of LMWH, followed by warfarin to attain an INR of 2–3 for six months or warfarin for life in cases of recurrent VTE. This regime is very effective in patients without cancer. However, the use of vitamin K antagonist to treat thrombosis in cancer patients is associated with 3-fold increase in the risk of recurrent VTE and up to 6-fold increase in the risk of major bleeding in comparison with non-cancer patients [1]. Furthermore, difficulties in maintaining the international normalized ratio (INR) within the therapeutic range due to drug interactions, treatment interruption as a result of illness and invasive procedures (e.g. central line insertion) are common problems in patients with cancer. If warfarin have to be used in cancer patients with VTE for long-term anticoagulation, venous gangrene may be prevented by starting warfarin simultaneously with LMWH at small dose e.g. 5 mg, as large doses with lead to rapid increase in INR leading to depletion of protein C and subsequent venous gangrene [30].

A recent systemic review of clinical trails concluded that Low-molecular-weight heparin (LMWH) is modestly superior to unfractionated heparin at preventing recurrent DVT and is at least as effective as unfractionated heparin for treatment of pulmonary embolism [31]. LMWH is associated with a lower risk of bleeding, safe use in an outpatient setting without the need for laboratory monitoring and have a lower risk of heparin-induced thrombocytopenia. Interestingly, recent evidence from randomised trails (FAMOUS and CLOT trails) have suggested that LMWH may have anti-neoplastic effects and there is increased survival with its used in patients with advanced solid tumours [32,33].

In a case of established VG, warfarin should be stopped and LMWH should be started or continued. It is likely that LMWH provide potential promise as a safe and effective measure in the management of such patients [34]. Importantly, other measures such as limb elevation to reduce oedema and attention to skin care to prevent pressure sores, infection and wet gangrene cannot be overemphasised. In acute iliofemoral DVT venous thrombectomy, intrathrombus catheter-directed thrombolysis, and pharmacomechanical thrombolysis can be used to successfully remove venous thrombus with increasing safety [35] (table 3). On the other hand, in cases of proven HIT alternative anticoagulation such as direct thrombin inhibitors (DTI) e.g. hirudin or argatroban can be used [36] (table 2, 3).

**Table 3:** Management of patients with venous gangrene

**Investigations**:
• Full Blood Count looking at the platelets count
• INR
• D-dimers
• Anticardiolipin antibody levels
• Protein C & S levels
• Venous doppler/venography
• Arterial pulses (palpation or by hand-held doppler)

**Treatment**

• Stop warfarin
• Start LMWH or DTI is cases of proven HIT
• Limb elevation to decrease the swelling
• Good nutrition and hydration of the patient
• Treatment of the underlying malignancy
• Venous thrombectomy or intrathrombus catheter-directed thrombolysis

## Conclusion

Venous gangrene is rare in association with malignancy. The pathogenesis is complex and involves a series of different mechanisms. Hypercoagulable state associated with malignancy and associated increase or decrease in clotting factors, cancer treatment, prolonged immobilisation, surgical operations and metabolic syndrome are all associated with increased risk of VG. The current evidence suggests that LMWH is the drug of choice in the treatment of venous thromboembolism and gangrene. It is safe and effective and associated with lower risk of bleeding, fewer recurrent VTE, lower risk of HIT and venous gangrene in comparison with UFH and Vitamin K antagonists. Further research will reveal the complex mechanisms and provide evidence for safe and effective treatment.
